# Supplementary material for: Spotted hyaena space use in relation to human infrastructure inside a protected area
Source: PeerJ. 2016 Oct 19;4:e2596. doi: 10.7717/peerj.2596 (PMC5075705; doi:10.7717/peerj.2596)
Supplement: Supplemental Information 1 — To evaluate whether or not we had sufficient sample sizes to estimate seasonal home ranges we created accumulation curves for each clan and season. We created randomized sets of coordinates with increasing sample sizes from 10 relocations up to the actual sample size used for each seasonal range. For each sample size, we randomly drew 100 data sets without replacements from the original sets of coordinates that was utilized to calculate each seasonal home range, and for each random data set we calculated the area covered by a 100% MCP. These areas were plotted against sample size. [file peerj-04-2596-s001.pdf]

Supplementary Information 1

Estimation of convergence of home range size estimates

To evaluate whether or not we had sufficient sample sizes to estimate seasonal home ranges we created accumulation curves for each clan and season. We created randomized sets of coordinates with increasing sample sizes from 10 relocations up to the actual sample size used for each seasonal range. For each sample size, we randomly drew 100 data sets without replacements from the original sets of coordinates that was utilized to calculate each seasonal home range, and for each random data set we calculated the area covered by a 100% MCP. These areas were plotted against sample size.

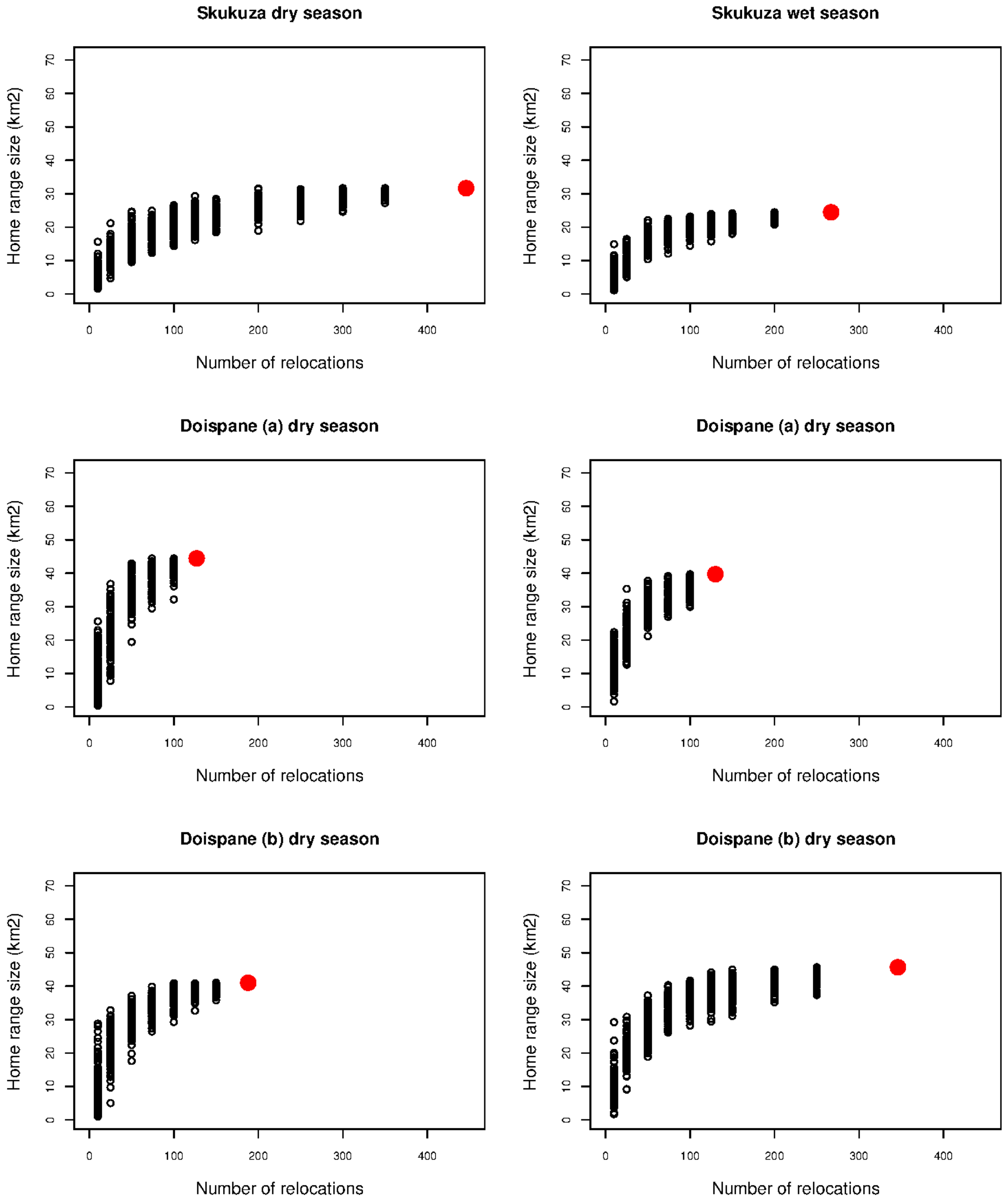

Fig. S1. Accumulation curves for home range size for each clan and season. The large red dot marks the estimated home range size given the full set of data that were utilized.
